# Throwing the baby out with the bath water — response to the Swedish Agency for Health Technology Assessment and Assessment of Social Services (SBU) report on traumatic shaking

**DOI:** 10.1007/s00247-017-3932-8

**Published:** 2017-08-07

**Authors:** Dawn Saunders, Maria Raissaki, Sabah Servaes, Catherine Adamsbaum, Arabinda Kumar Choudhary, Joëlle Anne Moreno, Rick R. van Rijn, Amaka C. Offiah, Written on behalf of the European Society of Paediatric Radiology Child Abuse Task Force and the Society for Pediatric Radiology Child Abuse Committee

**Affiliations:** 1 Great Ormond Street Hospital NHS Trust for Children, Institute of Child Health, WC1N 3JH, London, UK; 2 Department of Radiology, University Hospital of Heraklion, Iraklio, Greece; 3 University of Crete, Heraklion, Crete, Greece; 4 The Children’s Hospital of Philadelphia, University of Pennsylvania, Philadelphia, PA USA; 5 AP-HP, Bicêtre Hospital, Pediatric Imaging Department, Paris Sud University, Bicêtre, France; 6 Department of Medical Imaging, Alfred I. duPont Hospital for Children, Wilmington, DE USA; 7 Florida International University, College of Law, Miami, FL USA; 8 Department of Radiology, Emma Children’s Hospital, Academic Medical Center, Amsterdam, The Netherlands; 9 Academic Unit of Child Health, Sheffield Childrens NHS Foundation Trust, University of Sheffield, Sheffield, UK

The recent publication by Lynøe et al. [1] provides an opportunity to debate the diagnosis of abusive head trauma in scientific journals instead of behind the (often closed) doors of the courtroom. The Swedish Agency for Health Technology Assessment and Assessment of Social Services (SBU) report [2] casts doubt on the quality of the evidence of shaken baby syndrome, is cited in the French Wikipedia and has already been cited in court. The new report raises major medical concern because it may already have disrupted efforts to protect vulnerable children. Shaking is repeated in more than half of reported cases of abusive head trauma, and more than one child in the same family or household may be injured by the same perpetrator(s) in these cases. Recent efforts to cast unwarranted doubt on the medical fact that the diagnostic triad (subdural hematoma, cerebral edema and retinal hemorrhages) can reliably be associated with abusive head trauma may have catastrophic consequences. It should be noted that in contrast to the inexplicably narrow focus of the SBU panel, pediatric radiologists consider shaking as a possible — but not the only — form of physical abuse. As physicians, we do not diagnose *shaking*, we diagnose *abuse*. Neither do we diagnose *the triad* — which is a lawyer-created name for a constellation of medical findings that may have multiple generally possible causes but that in any specific case helps physicians who treat and diagnose infants and children to determine the most medically plausible explanation for head trauma injuries.

The paper by Lynøe et al. [1] constitutes a summary of the published SBU report of 2016 completed by the same authors [2]. The review states that, “*In cases of suspected traumatic shaking, the diagnosis has conventionally been based on three findings, referred to collectively as the triad, namely: subdural hematoma (SDH) (bleeding between the dura mater and the brain), retinal hemorrhages, and various forms of brain symptoms (encephalopathy)*.” This statement is both inaccurate and misleading. Although findings that include subdural hematoma, retinal hemorrhages, and various forms of brain symptoms (encephalopathy) would be sufficient for any physician to consider abuse, in most papers dealing with this topic (and all cases in clinical practice), an abuse diagnosis relies upon careful review of all available data, often including data identified and assessed by a dedicated multidisciplinary team, a constellation of imaging findings in the brain, bones, neck, spine and abdomen, fundoscopic findings, interviews with caregivers, forensic data (including postmortem studies), the presence of additional or previous injuries to the child or siblings, the presence of other malicious injury (e.g., burns, bite marks) and exclusion of underlying diseases and accidental injury. This rigorous diagnostic approach is in accordance with the recently published review of guidelines for the investigation of a child suspected of being physically abused [3].

In addition to their misleading characterization of the triad as the sole basis for every abusive head trauma diagnosis, Lynøe et al. [1] rely on an artificially constrained gold standard for abuse that requires that the inflicted injury be: "*admitted or witnessed traumatic shaking or other trauma.*" Their use of the term “other trauma” as part of their self-defined diagnostic gold standard is nebulous. More specifically, the omission of a detailed definition of “other trauma” creates additional confusion and doubt about their methods and conclusions.

The problems created by the insertion of a new narrow diagnostic gold standard are compounded by the authors’ skepticism regarding confession evidence, which they deem to have a high risk of bias. According to Lynøe et al. [1]: “Confessions are difficult to obtain and may not always be reliable.” Numerous papers dealing with abuse in the medical literature include cases accompanied by confessions or convictions by a coroner/judicial professionals, based on the combination of evidence and its scientific plausibility and consistency [4–12]. However, Lynøe et al. [1] simply decided to reject all but two of these papers [11, 12] because they deemed the bulk of the medico-legal literature — in which confession evidence consistent with medical findings helped to confirm the diagnosis of abuse — to be of low quality. According to the authors, these papers were excluded from their meta-analysis because there were no detailed descriptions of the circumstances of the confessions [4–10]. The authors also ignored papers describing forensic pathology findings in fatally abused children, where confessions *were* available, because they deemed this research to be of low quality, despite the fact that physicians generally accept forensic pathology research to be a genuine gold standard of empirical science [9, 13–15]. According to the authors, only a single paper [12] had an acceptable control group for inclusion in their study.

For obvious reasons, witnessed or videotaped abuse and fully detailed confessions are very rarely available to be included in the medical literature or as a part of clinical practice. In fact, this is partially explained by the papers rejected by Lynøe et al. [1], which show that perpetrators often underestimate the degree of injury (most likely because they recognize the socially reprehensible nature of the act or in an attempt to limit their criminal punishment) [16, 17]. As global evidence-gathering efforts continue to improve, overlapping injury patterns and consistent after-the-fact statements from perpetrators will continue to demonstrate that confession evidence can help to establish the cause of inflicted head injuries [4–12, 16–23], including one case in which the medical evidence for shaking is compelling and the details of how the confession was made are provided in stark detail [21].

Given the difficulty of obtaining confessional evidence, Lynøe et al. [1] rejected the obvious and more appropriate methodology for their study — the use of a control group cohort of children with one or more components of the triad, who were given other diagnoses (e.g., accidental injury, infection, metabolic conditions). Radiologists do not conclude abuse merely because of the presence of the triad. Other conditions are always excluded either *before* or *following* referral to the child protection team. The SBU methodology is fatally problematic because it disregards this basic but essential component of the diagnostic process. The authors’ failure to recognize these analytic flaws may be the result of the lack of expertise among the SBU panel when it comes to evaluating these cases. The SBU methodology purports to rely solely on a cohort of children with triad findings who were referred to a child protection team, but the authors totally ignore every child who presented with any component of the triad but were not referred to a child protection team (Table 1 in the SBU report [2]).

We also have doubts regarding the validity of the population, index test, reference test/gold standard and outcome (PIRO) as implemented by Lynøe et al. [1]; P (children ≤12 months of age), I (the triad in cases of suspected traumatic shaking), R (admitted or witnessed traumatic shaking or other trauma) and O (diagnostic accuracy). We are curious as to how the authors assessed the diagnostic accuracy of the index test (triad) and reference test (confession) — i.e. what was the reference used? If the question is how reliable is the triad in detecting shaking, then we believe a more robust PIRO would be P (children ≤12 months of age), I (the triad in cases of suspected traumatic shaking), R (the triad in cases of diagnoses other than suspected traumatic shaking), O (confession of shaking) [24]. This would identify critical information, i.e. how many cases of confessed shaking do *not* result in the triad or any of its components.

In practice, radiologists do not distinguish shaking from abuse, per se, and do not isolate intracranial from other injuries (e.g., metaphyseal and rib fractures). In fact, a radiologist, ophthalmologist, forensic pathologist or pediatrician who would deliberately blind himself/herself to relevant medical information would be unable to meet the standard of care. Despite this fact, Lynøe et al. [1] rejected 28 relevant papers [4–10, 13–15, 25–42] because they were deemed to be scientifically limited or insufficient regarding the diagnostic accuracy of the triad in the identification of traumatic shaking. These papers were rejected despite the fact that many [5–8, 10, 14, 25–34, 36–42] included children with injuries at multiple sites, the combination of which would cause any practicing radiologist to suspect abuse. According to the authors, these papers were unworthy of consideration because they purportedly included risk of circularity, lack of confessed cases, mixed groups of confessed and verdict-diagnosed cases, and selection and group allocation bias. Notably, this critique appears to have been directly borrowed from the lawyers and law professors who defend individuals accused of child abuse: “The validity of the research, therefore, depends entirely on whether researchers are accurately identifying which cases reflect abuse and which do not. But often the very diagnostic signs that are used to sort cases into these two categories are the same signs that the studies are purporting to measure; the research suffers from a circularity problem. As a consequence, most studies probably over-count the number of cases that are intentional, that are inflicted abuse. That is to say, the studies suffer from selection bias, observer bias, or both” [43].

The panel composition, search strategy, methodology, objectivity, transparency, accuracy, use of the generic term “retinal hemorrhage” without further descriptions of diagnostic significance and lack of explanations regarding the inclusion and exclusion criteria that Lynøe et al. [1] relied upon have already been the subject of some debate [44–48]. This may have been anticipated by the authors who twice caution, "It is important to note that limited evidence for the reliability of a method or an effect does not imply complete lack of scientific support” [1]. This suggests that they accept that the triad and therefore its components can be reliably associated with traumatic shaking [1]. Of note, they do not concede this in their review paper and only repeat their views regarding the diagnostic accuracy of the triad in the identification of shaking (which their methodology has not addressed).

Finally, in an attempt "to determine the diagnostic accuracy of the triad in detecting that an infant has been violently shaken" [1] and "to determine how reliably the triad or its components can be explained by traumatic shaking of children up to one year of age" the authors failed to incorporate the article of Biron and Shelton [23], who described this association in confessed cases of shaking. Additionally, Barlow et al. [25] described a child who presented with “the triad” alone, was sent home and later presented with metaphyseal fractures and bruising of the buttocks (case 9 of that report). These cases appear sufficient to support the assumption that when “the triad” is present, abuse needs to be excluded. This also supports our impression that the questions posed were not answered using the methodological approach employed by the review and set out in the SBU report (described as PIRO).

The SBU report raises the following issues: the need for collaboration among health specialists and judicial professionals; and the need for gathering detailed descriptions/information on imaging, funduscopic appearances, forensic data, presenting history, caretaker interviews, and medical data of children abused beyond any doubt, that is based on reliable and detailed confessions, and storing these in an international databank for research, training and medico-legal purposes.

We welcome the call for future research and international coordination and recommend that we start by developing consensus-based definitions and pro forma for uniform recording of data associated with abuse. This will go some way to standardizing the conduct and reporting of research in this field. Meanwhile, radiologists should remain vigilant in their daily practice. Small subdural collections, imaging evidence of brain edema, ischemia, hemorrhagic lesions inside the brain, hematomas in the soft tissues, skull fractures and spinal abnormalities should all be carefully looked for, documented and reported in detail *together* with other evidence of disease or trauma elsewhere, ensuring an unbiased approach to the identification and recording of medical evidence supporting either abuse or alternative diagnoses.

“Don't throw the baby out with the bathwater” is an idiomatic expression and a concept used to suggest an avoidable error in which something good is eliminated when trying to get rid of something bad, or in other words, rejecting the essential along with the inessential [49]. The idiom is applicable when someone might throw out the baby and keep the bathwater [50].

The SBU report, possibly attempting to protect people falsely accused of perpetrating abuse, is likely to achieve an unacceptable end: providing lawyers with new ammunition to question valid scientific data. As shown, this ammunition relies on a methodologically flawed review of the evidence and on the exclusion of all information inconsistent with the conclusions set forth by Lynøe et al. [1]. As recently stated by Judge Richard L. Bucher of the New York Supreme Court, “the anti-SBS diagnosis” is espoused only by a distinct minority of physicians who believe “that the only way to respond to medical evidence was to present counter medical opinions” with “no weighing of the strengths and weaknesses of the different strategies on the basis of the medical and other evidence” [51]. This is the approach of the SBU report, which left unchecked will result in failure to protect abused and vulnerable children.
